# Poly-ε-Caprolactone Implants for Benznidazole Prolonged Release: An Alternative to Chagas Disease Oral Treatment

**DOI:** 10.3390/pharmaceutics15041126

**Published:** 2023-04-02

**Authors:** Ana Lia Mazzeti, Karolina R. Gonçalves, Patrícia Ferreira Boasquívis, Jamile Barbosa, Bruno G. Pereira, Maria de Nazaré Correia Soeiro, Vanessa Carla Furtado Mosqueira, Maria Terezinha Bahia

**Affiliations:** 1 Laboratório de Biologia Celular, Instituto Oswaldo Cruz, Fundação Oswaldo Cruz, Rio de Janeiro 21040-360, RJ, Brazil; 2 Laboratório de Parasitologia Básica e Aplicada, Universidade do Estado de Minas Gerais, Unidade Acadêmica de Passos, Passos 37900-106, MG, Brazil; 3 Laboratório de Doenças Parasitárias, Escola de Medicina & Núcleo de Pesquisas em Ciências Biológicas, Universidade Federal de Ouro Preto, Ouro Preto 35400-000, MG, Brazil; 4 Diretoria Industrial, Fundação Ezequiel Dias, Belo Horizonte 30510-010, MG, Brazil; 5 Laboratory of Pharmaceutics and Nanotechnology (LDGNano), School of Pharmacy, Federal University of Ouro Preto, Ouro Preto 35400-000, MG, Brazil

**Keywords:** *Trypanosoma cruzi*, benznidazole, implants, Chagas disease, drug delivery, biodegradable polymer

## Abstract

Benznidazole (BZ) tablets are the currently prescribed treatment for Chagas disease. However, BZ presents limited efficacy and a prolonged treatment regimen with dose-dependent side effects. The design and development of new BZ subcutaneous (SC) implants based on the biodegradable poly-ɛ-caprolactone (PCL) is proposed in this study for a controlled release of BZ and to improve patient compliance. The BZ–PCL implants were characterized by X-ray diffraction, differential scanning calorimetry, and scanning electron microscopy, which indicated that BZ remains in its crystalline state dispersed in the polymer matrix with no polymorphic transitions. BZ–PCL implants, even at the highest doses, induce no alteration of the levels of hepatic enzymes in treated animals. BZ release from implants to blood was monitored in plasma during and after treatment in healthy and infected animals. Implants at equivalent oral doses increase the body’s exposure to BZ in the first days compared with oral therapy, exhibiting a safe profile and allowing sustained BZ concentrations in plasma to induce a cure of all mice in the experimental model of acute infection by the Y strain of *T. cruzi*. BZ–PCL implants have the same efficacy as 40 daily oral doses of BZ. Biodegradable BZ implants are a promising option to reduce failures related to poor adherence to treatment, with more comfort for patients, and with sustained BZ plasma concentration in the blood. These results are relevant for optimizing human Chagas disease treatment regimens.

## 1. Introduction

Chagas disease or American trypanosomiasis is a neglected disease that affects more than 6 million people worldwide and is caused by the parasite *Trypanosoma cruzi* [1]. The population at high risk is usually located in poorer rural conditions. Most infected people do not have access to early and appropriate diagnosis and treatment. Health issues associated with Chagas disease, such as reduced worker productivity and high mortality, pose a significant economic burden in the affected countries. It represents a loss of more than half a million years of disability-adjusted life and an annual cost of several billion dollars [2].

The nitro-heterocyclic drugs, benznidazole (BZ), and nifurtimox have been employed in Chagas disease therapy for almost 50 years and are the only ones currently recommended by the World Health Organization [1]. The treatment is effective in the early phase of the disease but presents variable and limited efficacy in the later chronic phase. The standard clinical protocol for adult patients is BZ at 5–7 mg/kg/day, given orally in two or three administrations, for 60 days, with a maximum dose of 300 mg/day [3,4]. This long treatment schedule as well as the high doses can lead to many side effects and frequent treatment interruption [5]. Although the scientific benefits of BZ treatment have been demonstrated in many countries, chemotherapeutic management of the disease is already controversial, because long treatment regimens cause low patient compliance [5,6].

Despite the large number of molecules evaluated experimentally in vitro, few new compounds were able to interfere with the infection or induce parasitological cure in animal models or clinical trials [7]. Different studies have investigated strategies to improve the efficacy and/or reduce the toxicity of treatment for Chagas disease. They consist of optimization of treatment regimes, combination therapy, and new formulations [8,9]. Lower doses of BZ, alone or combined, have been tested experimentally [10,11,12,13,14] or in clinical trials (BENDITA—NCT03378661, MultiBenz—NCT03191162) and highlighted the hypothesis that treatment with lower doses or for a shorter period may be as effective as the standard doses [15].

In this regard, new formulations with anti-*T. cruzi* drugs could improve treatment by changing the pharmacokinetic and pharmacodynamic profiles and delivering suitable drug concentrations with lower toxicity. Several pharmaceutical formulations have been designed to increase the dissolution rate of BZ by oral route, including microcrystals [16], solid dispersions [17], complexation with cyclodextrin [18], and polymeric [19] and lipid formulations [20,21].

Given the need for long-term oral treatment with conventional drugs for Chagas disease, the incorporation of BZ in polymeric subcutaneous implants would be of therapeutic value. This makes it possible to evaluate a new route of administration for the chemotherapy of Chagas disease, which could promote greater patient comfort, reduce or prevent BZ hepatic metabolism, and maintain sustained concentrations of BZ in plasma. Sustained plasma concentrations can also contribute to reducing adverse effects. Subcutaneous and biodegradable polymeric implants are indicated when long-term plasma concentrations are desired or when frequent administration is required [22,23]. These systems can be implanted without surgery or with low-risk outpatient surgery, allowing the controlled and/or prolonged release of the drug [24,25,26,27,28]. They are widely used for the administration of hormones and tested in the treatment of diseases of a variety of etiologies, from cancer [24,28,29], to HIV [30] and parasitic diseases [25,31,32].

The biodegradable poly-ε-caprolactone (PCL) is a synthetic polymer constantly investigated for controlled or prolonged drug delivery in different devices [32,33]. This polyester has been approved by the FDA, presents a slow rate of degradation, and is low-cost. Thus, this work aimed to develop and characterize PCL implants incorporating BZ (BZ–PCL), evaluate the efficacy and toxicity, and determine in vivo release by quantifying the plasma concentration–time profiles of BZ delivered by these devices in an experimental model of acute infection by the Y strain of *T. cruzi* in mice.

## 2. Materials and Methods

### 2.1. Drugs and Materials

BZ, (N-benzyl-2-nitro-1-imidazolacetamide) was provided by Laboratório Farmacêutico do Recife (LAFEPE, Pernambuco, Brazil). Omeprazole (6-methoxy-2-[(R)-(4-methoxy-3,5-dimethylpyridin-2-yl)methylsulfinyl]-1H-benzimidazole) was purchased from Merck/Sigma-Aldrich (St. Louis, MO, USA). Cyclophosphamide (*N,N*-bis(2-chloroethyl)-1,3,2-oxazaphosphinan-2-amine 2-oxide) (Genuxal^®^) was purchased from Asta Medica Oncologica (Germany). The solvents were all of the analytical grade or HPLC grade. HPLC-grade acetonitrile, acetone, and ethyl acetate were provided by Tedia^®^ (Rio de Janeiro, Brazil). Poly-ε-caprolactone polymer (PCL; Mw 65,000 and Mn 42,500 g/mol, Ð 1.529 at 25°C) was purchased from Sigma (Sigma-Aldrich Co., St. Louis, MO, USA). We purified the water used throughout the experiments with the MilliQ^®^ system (Symplicity System 185, Millipore, Burlington, MA, USA).

### 2.2. Preparation of PCL Implants Containing Benznidazole (BZ–PCL)

The implants were prepared at different ratios of the PCL: BZ (1:0.66; 1:1; 1:1.33 *wt*/*wt*). The solid implants were molded in semicircle glass molds by the casting method. The BZ doses incorporated into the PCL polymer were defined according to the standardized dose for the oral treatment of mice (100 mg/kg) with an average body weight of 25 g (Table 1). BZ was weighed in the glass molds (25, 50, or 75 mg), and 100 μL of acetone and PCL (75 mg) were added. The mixture was stirred and heated (65 °C) in a water bath under 50 rpm agitation until complete dissolution. The blend was maintained under heating at 65 °C until complete evaporation of acetone and implant solidification under a chemical hood. The implants were then submitted to residual solvent removal for 96 h under reduced pressure in a desiccator. Implants without BZ (blank implants) were also prepared. Thus, 20-, 30-, and 40-fold the daily dose of BZ per mouse was incorporated into PCL implants. The resulting implants were semi-circle shaped, 10 mm in diameter.

### 2.3. Characterization of PCL Implants Incorporating Benznidazole (BZ–PCL)

Implants with the highest content of BZ (BZ–PCL 3), i.e., 75 mg of PCL and 100 mg of BZ (Table 1) were characterized by X-ray diffraction analysis (XRD), scanning electron microscopy (SEM), differential scanning calorimetry (DSC) and thermo-optic analysis (TOA).

#### 2.3.1. X-ray Diffraction Analysis (XRD)

XRD patterns of BZ, PCL implants (without drug), and BZ–PCL3 were obtained using an X-ray diffractometer Rigaku Miniflex 300 (Japan). The diffractogram was collected at an angle range from 2° to 45° 2θ, 30 kV, and 10 mA, with increments of 0.02° and a reading speed of 5°/min, and high-speed detector model D-Tex Ultra. Physical blends of BZ and PCL in the same proportions (1:1.33) and concentrations were prepared in parallel and analyzed.

#### 2.3.2. Scanning Electron Microscopy (SEM)

SEM was performed using a JEOL microscope JCM—6000 (Japan). Samples of BZ, PCL implants (without drug) and BZ–PCL3 (1:1.33) were prepared on double-sided carbon tape and fixed on an aluminum stub. Before the microscopical examination, pure drug samples were sputter-coated with a gold layer (Denton Vacuum, model Desk V). Images were obtained at 15 kV.

#### 2.3.3. Differential Scanning Calorimetry (DSC)

DSC thermograms of BZ, PCL implants (without drug) and BZ–PCL3 (1:1.33) were obtained on DSC 822e equipment (Mettler-Toledo, Schwerzenbach, Switzerland) and verified with zinc and indium standards. Samples (about 2 mg) were prepared in aluminum pans. The temperature ranged from 25–220 °C with a heating rate of 10 °C/min under a nitrogen atmosphere. No event related to the solvent used to prepare implants (acetone) was identified.

#### 2.3.4. Thermo-Optic Analysis (TOA)

TOA analyses of BZ and a physical blend of PCL/BZ (1:1.33) were performed using Mettler Toledo FP900 equipment, with an FP90 processor and FP82 hot stage (Mettler-Toledo, Schwerzenbach, Switzerland). Images were captured by Leica DM4000B optical microscope (Wetzlar, Germany) at 100×. Pure BZ samples were analyzed in the temperature range of 175 to 215 °C, at a heating rate of 2 °C/min. The physical blends of PCL and BZ samples were analyzed in the temperature range of 55 to 75 °C for PCL and 180 to 205 °C for BZ at a heating rate of 2 °C/min.

### 2.4. Animals and Ethical Concerns

We maintained female Swiss mice weighing from 18–24 g at 22 ± 2 °C at the animal facility of the Federal University of Ouro Preto, Minas Gerais, Brazil, in a controlled temperature room with access to water and food *ad libitum* under 12 h day/night cycles. All procedures related to animal use conformed with the Ethical Principles of Animal Experimentation of the Brazilian College of Animal Experimentation (COBEA), and all procedures and experimental conditions were approved by The Ethics Committee in Animal Research at UFOP under the number 2015/54.

### 2.5. Anti-T. cruzi Efficacy Experiments

**Figure 1:**
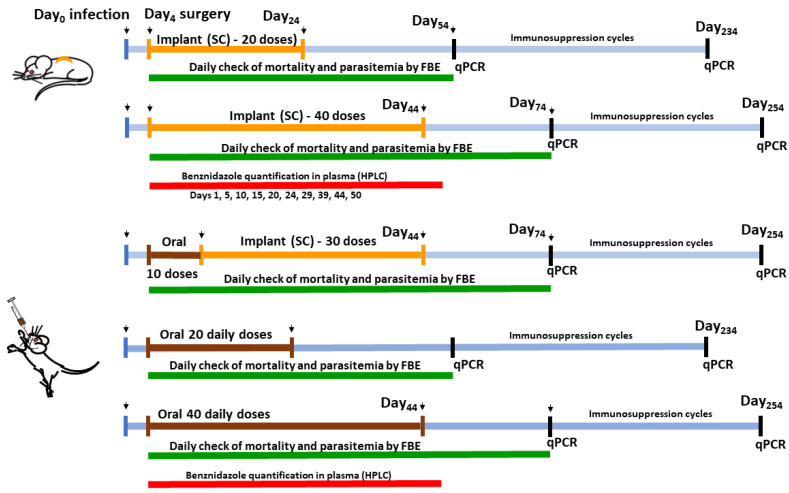
Experimental design of in vivo experiments of efficacy and determination of benznidazole plasma concentration–time profiles during treatment with the different benznidazole formulations (oral doses and subcutaneous implants).

To determine efficacy in an experimental murine model of infection, we inoculated the mice by intraperitoneal route with 5.000 trypomastigotes of the Y strain of *Trypanosoma cruzi*. This strain is partially sensitive to BZ treatment [33]. Treatments (gavage or SC surgery for implants) started on the 4th day of infection. Animals were anesthetized intraperitoneally with a mixture of xylazine (10 mg/kg) and ketamine (100 mg/kg). We shaved the hair in the dorsal region closest to the neck of the mice and then asepsis was performed with a 70% ethanol solution before surgery for subcutaneous implantation of the polymeric implants. We made a linear incision and divulsion with artery forceps to produce a subcutaneous pouch where the implants were introduced. Implants with BZ (BZ–PCL) and without BZ (blank-PCL) were tested. After the implantation surgery, the animals were maintained in individual cages with access to food and water *ad libitum*. Subsequently, the animals were monitored for any signs of infection at the operative site, or upon discomfort or distress. Any mice presenting such signs were immediately euthanized. Complete healing at the site of surgery was observed in all animals. We also treated a control group of mice by oral route with 100 mg/kg/day of BZ crushed tablets suspended in 0.5% (*wt*/*v*) methylcellulose and administered by gavage. The experimental groups are shown in Table 1 and in the schematic experimental design in Figure 1.

The animals were examined at least once a day to observe the occurrence of mortality and changes in clinical signs: appearance (twisting, piloerection, dirty eyes), pain (torsion, spasm), and behavior changes (withdrawal, vocalization, scratching, reluctance to move, irritability, anorexia, abnormal posture, ataxia).

### 2.6. Cure Control Tests

The efficacy of the treatment was estimated by detecting parasites by fresh blood examination (FBE), qPCR, and mortality, before and after the immunosuppression cycles, determined following the protocol previously described [34]. Briefly, mortality was checked daily until 30 days after treatment. Fresh blood examination according to the Brener method [35] was performed daily to estimate parasitemia during and up to 30 days after the end of treatment. Animals with negative results in the fresh blood examination were immunosuppressed with 50 mg/kg/day of cyclophosphamide (Baxter Oncology, Bielefeld, Germany) by intraperitoneal route in 3 cycles of 4 consecutive daily doses with an interval of 3 days between each cycle. The parasitemia was checked daily during and up to 10 days after the end of the immunosuppression cycles (Figure 1).

Blood qPCR analyses were performed 30 and 180 days after the end of the treatment in samples from mice with negative fresh blood examination results. Promega Wizard genomic DNA purification kit (United States) was used to extract the genomic DNA of 200 μL of blood samples, according to the manufacturer’s instructions. PCR analyses were performed using these primers: TCZ-F (5=-GCTCTTGCCCACAMGGGTGC-3=, where M indicates A or C) and TCZ-R (5=-CCAAGCAGCGGATAGTTCAGG-3=), as described by [36]. The presence of *T. cruzi* in blood samples was evaluated by amplifying a 195 bp tandem repeat in genomic DNA [36]. The murine TNF-α gene sequence was amplified separately using the primers TNF-5241(5=-TCCCTCTCATCAGTTCTATGGCCCA-3=) and TNF-5411 (5=-CAGCAAGCATCTATGCACTTAGACCCC-3=) [36]. For reactions, 2 μL template DNA at 25 ng/µL, and specific primers at concentrations of 10 μM and 5 μL of Sybr-Green PCR Master Mix (Applied Biosystems) in a total volume of 10 μL were used. DNA amplifications were carried out in 7500 Fast Real-Time PCR Systems (Applied Biosystems). After the initial denaturation step of 10 min at 95 °C, amplifications were carried out for 40 cycles (94 °C for 15 s). Fluorescence data collection was performed at 64.3 °C for 1 min at the end of each cycle. Amplification was immediately followed by a melting program with initial denaturation for 15 s at 95 °C, cooling to 60 °C for 1 min, and then stepwise temperature increases from 60 to 95 °C at 0.3 °C/s. All samples were analyzed in duplicate, and negative samples and reagent controls were processed in parallel in each assay. Animals showing negative results in all tests were considered cured.

### 2.7. Hepatic Toxicity Evaluation

The toxicity of treatments was evaluated by hepatic enzyme dosages in mouse serum collected on the last day of treatment. Aspartate aminotransferase (AST) and alanine aminotransferase (ALT) were determined by colorimetric assay using an autoanalyzer (Wiener Lab model CM200—kinetic analysis) and commercial Bioclin^®^ kit according to the manufacturer’s instructions.

### 2.8. Benznidazole Plasmatic Concentration following Time

We collected blood samples of mice at 1, 5, 10, 15, 20, 24, 29, 39, 44 and 50 days after the beginning of the treatment with implants or oral BZ. We quantified BZ in the plasma of infected (n = 3) and uninfected mice (n = 3) treated with the implant of BZ–PCL3 or treated for 40 days with oral BZ at the same final total dose (100 mg/kg/day). Blood samples were centrifuged after collection and the plasma was separated for further extraction. Omeprazole internal standard was added to each blood sample, and liquid–liquid extraction was performed with ethyl acetate. The BZ extraction method and chromatographic conditions were performed according to [37]. Samples were analyzed by the high-performance liquid chromatography (HPLC) system Waters Alliance e2695 (Waters, Manchester, UK) with a UV detector (Waters 2489). A Phenomenex^®^ C18 column, Gemini NX (150 mm × 4.6 mm, 5 μm), preceded by a pre-column C18 Phenomenex AJO-7597 (2 mm × 4.6 mm, 3 μm) in a column oven at 40 °C was used in the analysis. The mobile phase was acetonitrile:water (30:70, *v*/*v*), pumped with the isocratic flow (1.0 mL/min). Detection was monitored at 324 nm, and the injection volume was 20 µL.

Non-compartmental analysis of plasma concentration–time data after extravascular input was performed. AUC_0-t_ was estimated using a trapezoidal linear up/Log down method using PK solver 2.0 add-in software [38].

### 2.9. Statistical Analysis

Statistical treatment of the data was performed using Graph Pad Prisma 5.01 statistical software (GraphPad Software Inc., San Diego, CA, USA). Results were expressed as mean  ±  standard deviation. Parametric data were analyzed with Student’s *t*-test and nonparametric data with the Mann–Whitney test for toxicity analysis. Statistical significance was established with 95% confidence intervals and *p*  <  0.05.

## 3. Results

The BZ crystals used in the implant formulation were characterized by XRD and DSC analysis. BZ–PCL implants were prepared by blending pure BZ with acetone (b.p. 56 °C) at 50 rpm agitation speed, followed by the addition and dissolution of PCL. The mixture melted at approximately 65 °C in a water bath. The implants were further characterized through XRD, SEM, DSC, and TOA for understanding BZ–PCL 3 structure and the interactions between its components.

**Figure 2:**
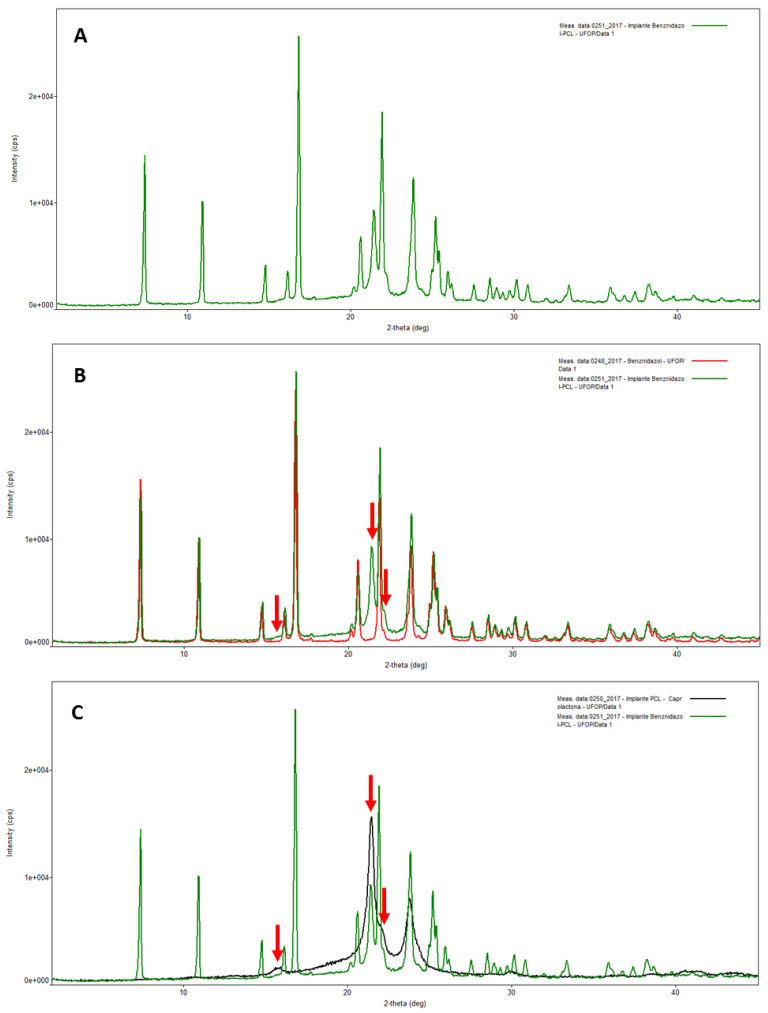
X-ray diffractogram of implants. (**A**) BZ–PCL implants. (**B**) BZ–PCL implants (green) and benznidazole (red). Red arrows indicate peaks that cannot be attributed to the pure drug. (**C**) BZ–PCL implants (green) and PCL implants (black). Red arrows indicate peaks that can be attributed to the diffraction profile of the implant without BZ.

The pure BZ XRD pattern revealed the most intense diffraction peaks at angular positions of 7.3697°, 16.7852° and 21.8729° 2Ɵ (Figure S1—Supplementary Information). Data refinement by the Rietveld method (PDXL2 software—Rigaku) using crystallographic information deposited by Soares-Sobrinho et al. [18] showed high similarity between the experimental and theoretical patterns (χ2 = 1.9145) (Figure S2 Supplementary Information), suggesting that the BZ experimental pure drug used in this study is the polymorphic form I. The blank-PCL implant (without BZ) XRD powder pattern (Figure S3—Supplementary Information) revealed its semicrystalline character, as only a few peaks at 15.8°, 21.4°, 23.8°, 30.2° and 40.9° 2Ɵ at angular positions were observed. The XRD powder patterns of BZ–PCL 3 implant and its comparison to BZ (pure) and blank-PCL implant are shown in Figure 2A–C, respectively. All diffraction peaks detected in the BZ–PCL 3 implant could be directly related to the peaks observed in the analysis of its isolated components. These data indicate that both drug and polymer remained in their crystalline and semi-crystalline states, respectively, after the implant preparation, suggesting that the BZ–PCL implant method of production did not promote the polymorphic transition of the BZ nor its change of crystalline to amorphous solid state.

**Figure 3:**
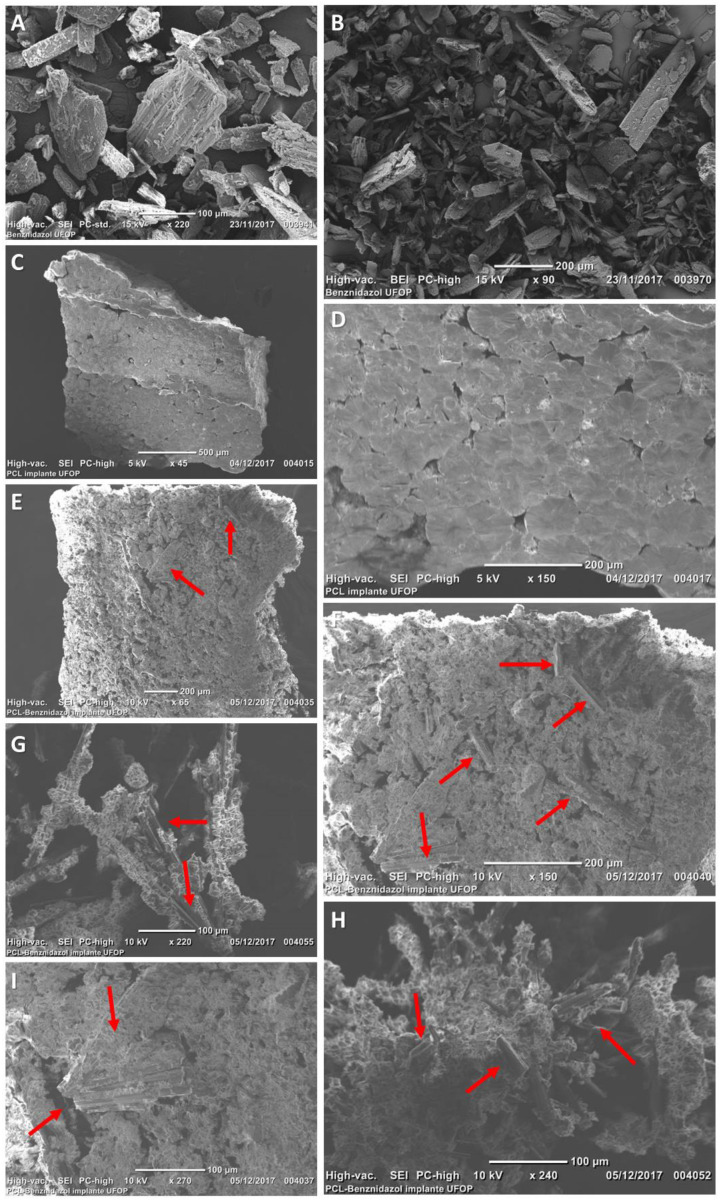
Scanning electron microphotographs of benznidazole: (**A**) 90× magnification; (**B**) magnification of 220×. PCL implants without drug: (**C**) 45× magnification; (**D**) 150× magnification. BZ–PCL implants at magnifications: (**E**) 65×; (**F**) 150×; (**G**) 220×; (**H**) 240×; (**I**) 270×. The red arrows indicate BZ crystals.

The implant characterization by SEM corroborated XRD results. The surface and cross-section morphologies of BZ (Figure 3A,B) and PCL implants (without drug) (Figure 3C,D) demonstrated BZ crystalline structure, while for the PCL implant, its porous characteristic stands out. Microphotographs of BZ–PCL3 implants showed a porous characteristic and the presence of intact BZ crystals, signaled by red arrows (Figure 3E–I), demonstrating that after implant preparation, the drug remained in its crystalline state, dispersed in the polymeric matrix.

**Figure 4:**
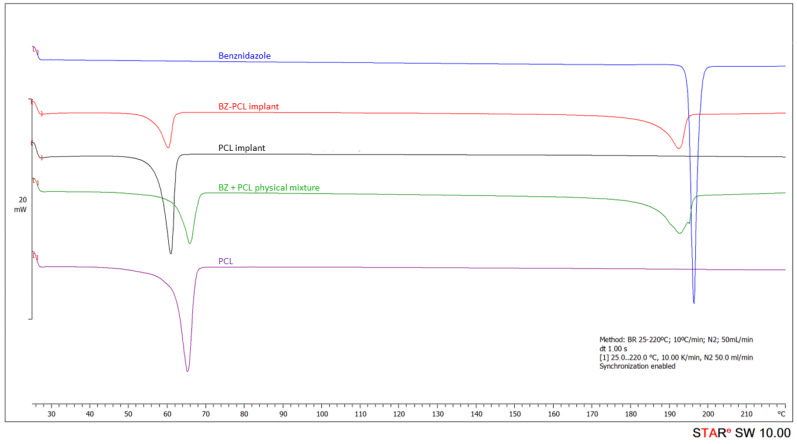
Differential scanning calorimetry (DSC) curves of BZ–PCL3 implant (ratio 1:1.33) (red), blank-PCL implant (black) and pure benznidazole (blue), physical mixture of PCL + benznidazole (1:1.33) (green) and PCL polymer (purple).

BZ and PCL interaction in the implant was further characterized by thermal analysis. Figure 4 presents the comparisons among DSC curves obtained for BZ (pure), blank-PCL implant and BZ–PCL implant. The BZ DSC curve showed an endothermic event ranging from 194.5 °C to 197.6 °C, with a peak at 195.2 °C and an enthalpy of −125.62 J/g (Table 2). This event corresponds to the fusion of BZ, observed between 188 °C and 199 °C., as previously reported by Soares-Sobrinho et al. [18]. A single endothermic event in the DSC curve of the PCL implant was observed between 57.3 °C and 62.3 °C, with a peak at 60.7 °C and enthalpy of −92.67 J/g (Table 2). This event corresponds to PCL polymer melting, compatible with data describing PCL melting peak at approximately 60 °C [25].

In the DSC curve of the BZ–PCL3 implant, two endothermic events were observed, related to PCL and BZ fusion events. No variation was observed in the range and temperature of the polymer melting peak in the BZ–PCL implant. Additionally, considering PCL concentration corresponding to 42.86% of BZ–PCL3 formulation, the enthalpy calculated for the polymer melting in the DSC curve of the BZ–PCL implants would be −39.7 J/g, which is very close to the observed −36.1 J/g enthalpy (Figure 4).

To investigate this possible drug–excipient interaction, analyses were carried out with samples of the pure drug and polymer, which were not submitted to any implant procedures. DSC curves of the physical mixture of BZ + PCL, pure BZ, and PCL polymer are shown in Figure 4, and Table 2 shows the data obtained from the DSC curves. First, the evaluation of the PCL fusion event in both analyses before and after the implant manufacturing process shows the PCL fusion (61.6 °C to 67.2 °C) was anticipated after the implant manufacturing process (PCL implant = 57.3–62.3 °C, with a peak at 60.7 °C). This variation may indicate that the polymer has a more heterogeneous semicrystalline structure after the implant preparation process [25].

The physical mixture of BZ and PCL presented a thermal profile similar to BZ–PCL 3 implants. There was no variation in temperature and enthalpy of the thermal event of the PCL polymer in the mixture (Table 2). Differently, the BZ fusion event in the mixture was anticipated and extended, and presented lower enthalpy than expected (Figure 4). The calculated enthalpy for BZ melting would be approximately −71 J/g, and the experimental value was −59.17 J/g (Table 2).

In the DSC characterization experiments, we did not detect events related to acetone traces in implants. Furthermore, the acetone boiling point (56 °C) is much lower than the melting of PCL at 65 °C. We performed additional RMN analysis of the BZ–PCL implants, and no signal of protons related to acetone appears in the RMN spectra.

**Figure 5:**
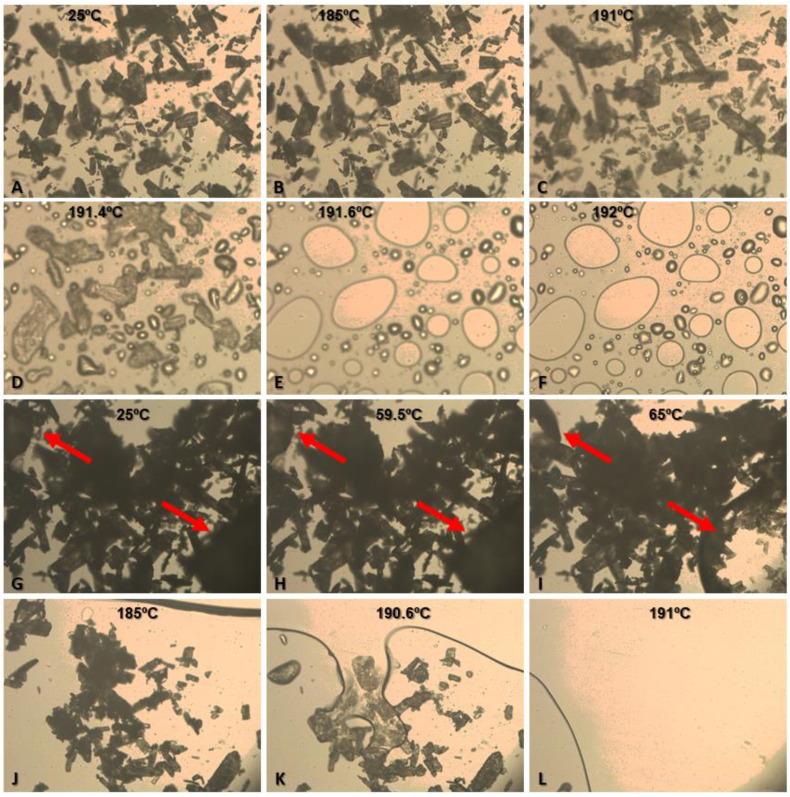
Thermo-optic analysis of benznidazole (**A**–**F**) and analysis of the physical mixture of PCL and benznidazole (**G**–**L**). The red arrows indicate particles of the PCL polymer.

To confirm the absence of interaction between the substances suggested by the DSC data, thermo-optic analysis (TOA) of BZ alone and a physical mixture of PCL and BZ (1:1.33) was performed (Figure 5). Under TOA conditions, BZ melting started at 191 °C, when the edges of the particles become less defined, and at 191.6 °C, the total melting of the sample particles was observed (Figure 5C–F). The variation of drug melting temperature in TOA analysis (191.6 °C) and in DSC analysis (t *_onset_* = 194.5 °C) could be explained by the analytical differences between techniques. In TOA conditions, samples are exposed to the ambient atmosphere, and the determination of thermal events is performed visually by the operator using an optical microscope. In the DSC analyses, samples are exposed to an inert atmosphere (nitrogen), and the determination of thermal events is performed automatically by the equipment software. Figure 5G–L shows the TOA data for the PCL + BZ mixture (1:1.33). The red arrows indicate PCL polymer particles (Figure 5G–I). The PCL polymer melting event started at 59.5 °C and extended until 65 °C, when no significant changes in the polymer melting were further observed (Figure 5H,I). At 185 °C, the polymer melting was spread in the observed field, and BZ particles were redistributed (Figure 5J). The fusion of the drug started at 190.6 °C, and all BZ crystals were completely fused at 191 °C, similarly to the pure drug (Figure 5K,L). Importantly, no darkening of the melt material was observed, suggesting that there was no degradation of the evaluated substances during these experiments.

Subsequently, the efficacy and toxicity of implant formulations were accessed in an experimental murine model of acute infection by *T. cruzi* Y strain (n = 6–8) [39]. Implants containing 75 mg or 100 mg of polymer were inserted in the subcutaneous tissue at the dorsum of uninfected mice. Animals showed rapid recovery from surgery and no signal of pain, discomfort, or loss of weight was observed. Approximately 60 days after implant insertion, an ulceration on the spot was observed in one animal (1 of 6) that receive a blank-PCL implant, and this mouse was euthanized. In this way, implants containing 75 mg of PCL and 50 (BZ–PCL 1) or 100 mg (BZ–PCL 3) of BZ were inserted, corresponding to 20 and 40 days of oral treatment, respectively (Table 3). A group was treated orally for 10 days with a daily dose of 100 mg/kg) and then had an implant inserted containing 75 mg of BZ (BZ–PCL 2), corresponding to 30 days of oral doses.. Other animals received 100 mg/kg/day of BZ by oral route for 20 or 40 days. Animals infected and untreated were the control group.

Treatments with subcutaneous BZ–PCL implants or with crushed tablets administered *per os* were able to prevent animal mortality, while the group of untreated and infected mice reached 100% mortality by the 18th day post-infection. Both treatments induced suppression of parasitemia at similar periods: 1.43 ± 0.77 days after oral administration for BZ and 1.20 ± 0.45 days after BZ implant SC insertion. Importantly, parasitemia reactivation was not detected in the interval of oral treatment and implant insertion. Oral standard treatment (100 mg/kg/day for 20 days) induced a cure in 57.1% (4 out of 7) of infected animals. When this same dose was administered via implant (BZ–PCL1), no cure was detected. In contrast, in 40 days of treatment with just an implant (BZ–PCL 3), or the combined protocol of 10 days by oral administration plus 30 days of the implant (BZ–PCL 2), 100% of the animals were cured (Table 3). In another report of our group, we observed 100% of cure with 40 doses of BZ administered by oral route [40].

**Figure 6:**
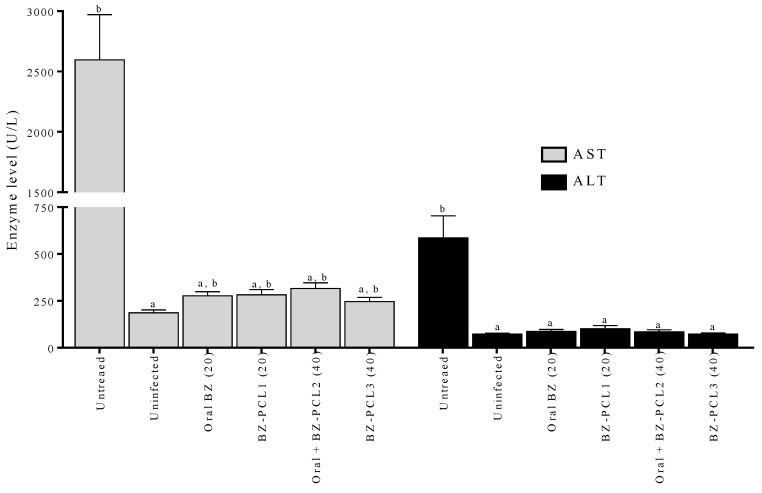
Levels of liver enzymes in the serum of animals treated with benznidazole. Levels of AST (aspartate aminotransferase) and ALT (alanine aminotransferase) detected in the serum of animals infected by strain Y, treated for 20 or 40 days with benznidazole. The samples were collected on the last day of treatment. The AST and ALT levels in untreated animals were measured on the 15th day of infection. *a* = significant difference from infected untreated; *b* = significant difference from uninfected untreated control.

Concerning the toxicological aspect of the implants and the possibility of induction of toxicity related to the high amount of BZ incorporated into the device, we evaluated the liver damage during treatment (Figure 6). The levels of liver enzymes AST and ALT were assessed in the animals’ serum on the last day of treatment. Figure 6 shows the levels of liver enzymes in the sera of animals treated with different regimens and formulations of BZ. Treatments significantly reduced the levels of AST and ALT in the animals’ serum compared with untreated infected animals. In addition, treatment for an extended period does not interfere with enzyme levels.

**Figure 7:**
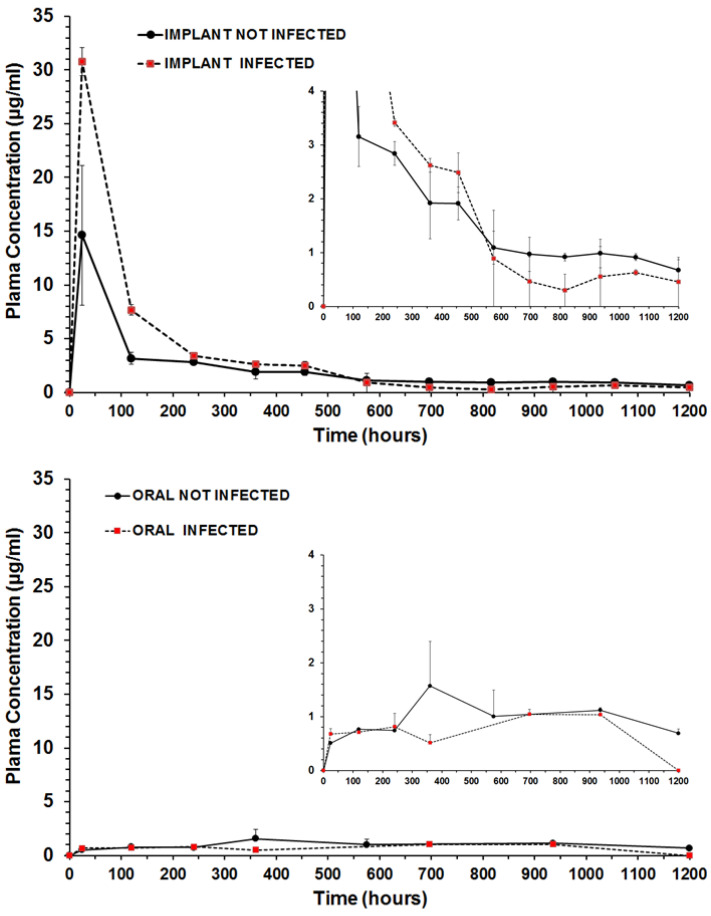
Benznidazole plasma concentration–time profiles upon administration of BZ–PCL implants (upper graph) and crushed tablet coarse suspension administered by gavage (lower graph), in healthy and *T. cruzi*-infected mice. Bars are standard deviations (n = 3 per group). Pharmacokinetic parameters were calculated and are shown in Table 4.

Figure 7 shows the profiles of plasmatic concentration of BZ following time during 40 days of treatment with oral doses (100 mg/kg/day) or with subcutaneous implant BZ-PLC3 in healthy mice or in *T. cruzi* infected mice. There is a clear difference (*p* < 0.05) between plasma exposure (area under the curve) after treatment with implants and oral administration. Implants maintain higher BZ plasmatic concentrations in the first days and a slower decay after five days compared with oral daily doses. These differences in BZ plasma concentrations are even higher in infected mice than in healthy mice. The disease plays a role in the body’s exposure to BZ with subcutaneous implant formulations. This effect is not clearly observed in the treatment by oral route.

Table 4 shows the pharmacokinetic parameters calculated using a non-compartmental model with extravascular input. Implants promoted a two-fold increase in the AUC for healthy and five-fold for infected mice compared with oral route. However, after 24 days of treatment, the plasma levels were similar for all formulations in healthy and infected mice (*p* > 0.05). Implants induce a burst effect in the first days, which guarantees high body exposure to BZ. Interestingly, the infection has an impact on BZ pharmacokinetics in mice. The body exposure decreased by 37% in the infected animals compared with healthy mice upon oral administration. Conversely, body exposure increased by 34% in infected animals treated with SC implants compared with healthy mice. This effect seems to be related to the differences in metabolism of BZ after oral and subcutaneous administration not evidenced up to date.

## 4. Discussion

BZ is a crystalline drug that can exist in three solid polymorphic forms: I, II, and III [41]. Form I is the most stable and the most available commercially. It has a melting point of around 190 °C [41,42,43] and a fusion range between 188 and 199 °C [41]. According to thermogravimetric (TGA) data, form I is thermally stable up to about 274 °C [41]. The other polymorphs were obtained through the recrystallization of polymorph I [41]. The water solubility of polymorphs I, II and III are close (0.22, 0.24, and 0.25 mg/mL, respectively), and they present similar XRD powder patterns, suggesting that differences in the crystalline packaging of the three crystalline forms are subtle [39]. PCL is a biodegradable hydrophobic polyester with high flexibility and elongation and tension strength [44]. In vivo degradation occurs by hydrolysis of ester bonds, and the slowness of this process makes the PCL suitable for implant formulation requiring long degradation and release periods [45,46]. PCL is a semicrystalline polymer, with glass transition at about−60 °C [25,41,44]. The analysis of our results indicates that PCL maintains its semi-crystalline form, and BZ maintains its polymorphic form I in the implants. Thus, the BZ–PCL implants are not a solid solution, and no strong interaction between the drug and PCL polymer was evidenced.

The analysis of DSC results suggests that the presence of the BZ did not change the polymer’s properties. Differently, the evaluation of the DSC curve of BZ thermal behavior in the PCL implant demonstrates a change in the fusion range: from 194.5 °C to 197.6 °C in the pure drug, to 186.7° to 194.6 °C in the BZ–PCL implant DSC curve. The observed fusion enthalpy (59.12 J/g) was lower than expected (about −71 J/g), considering the drug corresponds to 57.14% of the implant composition. These results suggest the occurrence of weak interaction between BZ and the polymeric matrix. Furthermore, analysis of TOA data suggests that there are no interactions between the PCL polymer and BZ. Nevertheless, stability studies must be performed to confirm that no chemical interactions occur between the substances after a longer period.

This work investigated whether the formulation of a subcutaneous polymeric deposit can release the drug in a continuous and controlled manner and bring benefits for the treatment of Chagas disease. The efficacy of BZ in experimental infection is influenced by dose, time, and route of drug administration [40,47].

Implants are charged with a high dose of BZ, and toxicological evaluation of liver eventual damage indicates no effect related to BZ. The levels of AST and ALT in the animals’ serum were reduced by controlling the infection and a reduction in tissue damage, possibly related to a decrease in the parasitic burden of the liver tissues. By contrast, Novaes et al. demonstrated that infection by the Y strain of *T. cruzi* and treatment with BZ increases the levels of liver enzymes ALT and AST compared to untreated and uninfected animals, indicating liver damage. These lesions may be the result of both BZ metabolism and specific mechanisms activated in *T. cruzi* infection [48]. The toxicity of BZ, due to its hepatic metabolism, and its low effectiveness, especially in the later chronic phase using the current treatment regimen of BZ, are factors that must be considered in the search for new alternatives.

Our data shows that BZ implants increase the plasma concentration of BZ compared with oral treatment with equivalent doses. Thus, an increase in BZ bioavailability with implants, mediated by reduced *first-pass* metabolism, can increase plasma and tissue concentrations. This 5-fold increase in bioavailability can lead to an increased incidence of adverse effects as well as increased efficacy against parasites in blood and tissues. Our results show that the treatment of animals infected by strain Y of *T. cruzi* using polymeric implants is probably similar to a longer regimen of 40 oral daily doses to produce a 100% cure in this strain. However, no adverse effect was observed with implants and no direct effect on hepatic biochemistry. Even though high concentrations of BZ detected at the beginning of treatment with BZ–PCL implants have the potential to cause some liver damage, after 20 or 40 days of implant insertion, no changes in AST and ALT enzymes were observed compared with treatment with oral BZ for 20 or 40 days. Together, our results show that the incorporation of BZ into polymeric implants is a promising alternative for the treatment of *T. cruzi* infections. Implants have been demonstrated to be safe in this study and their subcutaneous implantation does not alter the level of liver enzymes in the serum of treated animals. PCL polymer does not induce any signs of toxicity in the tissue.

The initial high plasma levels of BZ released by implants seemed to be necessary to clear parasites from blood and at the second step, particularly after 24 days of treatment, the lower but sustained plasma levels of BZ were probably responsible for eliminating tissue parasites and inducing 100% cure of infected animals. These results from 40-day treatment by oral or SC routes indicate that sustained plasma levels for a long time are required to clear parasites from the Y strain of *T. cruzi* in mice. Further improvements in BZ implant formulation are required to reduce the burst release in plasma in the first days after implantation.

Dormant/quiescent/persistent amastigotes are difficult to eliminate and may evade host immune surveillance, and their reactivation may result in drug failure; then, it has been suggested, that new anti-*T. cruzi* drugs or even BZ could be more efficacious under longer periods of administration or delivery [40]. Despite current efforts aimed at evaluating the optimization of dosing regimens, the dose is directly related to treatment efficacy in murine infection [40,47]. In this sense, new implant formulations can be developed, especially combinations of different polymers that can lead to a modulated release of BZ. This strategy may induce therapeutic efficacy with sustained BZ release at lower doses.

Thus, taken together, our results demonstrate that the route of administration, oral or subcutaneous (via implant) influences the body’s exposure to BZ from concentration–time plasmatic profiles. In the present study, efficacy was not significantly modified by both types of administration. However, implants prolong BZ release in the body and may improve the patient’s comfort. Biodegradable implants are devices representing an interesting option to increase compliance with treatment, ensuring the maintenance of the cure rates without the need for daily administration.

## 5. Conclusions

Chagas disease remains without an ideal treatment regimen for all phases of the infection. We developed and characterized, for the first time, implants for prolonged release of BZ as new promising devices for Chagas disease patients. These devices show the same efficacy as 40 oral daily doses in our experimental design. The implants, even with a high amount of BZ incorporated into the polymeric matrix, show a safe profile concerning hepatic toxicity. Importantly, these implants dramatically improved the plasmatic concentrations of BZ compared to oral treatment for 40 days, particularly in the first days after implantation. These new biodegradable BZ implants are an interesting option to reduce failures related to poor adherence to treatment with more comfort for patients and better optimization of human Chagas disease treatment.

## Figures and Tables

**Table 1:** Experimental groups and treatment schedules.

Treatment Schedule	Benznidazole Oral Dose (Days)	Benznidazole Implant Dose (Days)	Implant PCL:BZ Ratio	Benznidazole Total Dose/Per Mouse ^1^
blank-PCL implant	0 mg/kg	0 mg/kg	1:0	0 mg
BZ–PCL 1	0 mg/kg	100 mg/kg (20)	1:0.66	50 mg
BZ–PCL 2 ^$^	100 mg/kg (10)	100 mg/kg (30)	1:1	75 mg
BZ–PCL 3	0 mg/kg	100 mg/kg (40)	1:1.33	100 mg
Oral BZ ^&^	100 mg/kg (20)	0 mg/kg	-	50 mg
Oral BZ	100 mg/kg (40)	0 mg/kg	-	100 mg

^1^ BZ total dose at the end of treatment. Amount of drug incorporated into the polymer, corresponding to days of oral treatment with 100 mg/kg dose for a mouse.BZ: benznidazole. ^$^ Combined protocol: oral doses + implants. ^&^ Classical protocol of treatment with 20 doses of 100 mg/kg/day used in the efficacy assay against *T. cruzi*-infected mice.

**Table 2:** Comparative onsets, endsets, peak temperatures, and enthalpy of endothermic events determined from DSC curves (Figure 4).

Sample	DSC—PCL				DSC—Benznidazol			
	T*_onset_* (°C)	T_peak_ (°C)	T*_endset_* (°C)	Enthalpy (J/g)	T*_onset_* (°C)	T_peak_ (°C)	T*_endset_* (°C)	Enthalpy (J/g)
Benznidazole	--	--	--	--	194.54	195.19	197.62	−125.62
PCL polymer	61.59	64.83	67.18	−88.30	--	--	--	--
PCL implant	57.35	60.68	62.31	−92.67	--	--	--	--
BZ–PCL implant	56.35	60.19	61.80	−36.09	186.69	192.27	194.61	−59.12
Physical mixture BZ + PCL	62.11	65.65	68.08	−41.77	185.34	192.42	196.09	−59.17

**Table 3:** Efficacy of treatment with benznidazole subcutaneous polymeric implants and/or administered by oral route in the course of infection of mice infected with Y ^&^ strain of *Trypanosoma cruzi*.

Treatment Schedule/Groups	Nº of Daily Doses or Doses in Implants (mg/kg)	Parasitemia Suppression by FBE (Days)	Parasitemia Reactivation (Days) by FBE ^2^	Negative Total Tests ^3^
Uninfected	0	-/7	-	-/7 (100%)
Infected and untreated	0	0/7	-	0/7 (0%)
Implant BZ–PCL (20)	20 × 100 mg (implant)	5/5 (1.20 ± 0.45)	5/5 (22.8 ± 7.53)	0/5 (0%)
Oral + Implant BZ–PCL	10 × 100 mg (oral) + 30 × 100 (implant)	6/6 (1.17± 0.41)	0/6 (ND)	6/6 (100%)
Implant BZ–PCL3 (40)	40 × 100 mg (implant)	8/8 (1 ± 0)	0/8 (ND)	8/8 (100%)
Oral 100 mg/kg (20) ^1,@^	20 × 100 mg (oral)	7/7 (1.47 ± 0.77)	1/7 (27 ± 0)	4/7 (57.1%)
Oral 100 mg/kg (40) ^1,#,@^	40 × 100 mg (oral)	7/7 (1.47 ± 0.77)	0/7 (ND)	7/7 (100%)

PCL: poly-ε-caprolactone; BZ: benznidazole, FBE: fresh blood examination test; qPCR: quantitative polymerase chain reaction test in blood. ^@^ Crushed tablets in suspension administered by gavage. ^1^ *Swiss* mice (18–22 g) were inoculated with 5 × 10^3^ trypomastigotes of the *T. cruzi* Y strain. Treatment was started 4 days after infection and continued for 20–40 days. ^2^ Number of days for reactivation of parasitemia observed before immunosuppression. ^3^ Negative tests in fresh blood examination (FBE) before and after immunosuppression with cyclophosphamide and negative qPCR tests in blood were performed at 30 and 180 days after treatment. Animals showing negative results in all tests were considered cured. ^#^ Experiment was conducted simultaneously and the data have been reported previously [40]. ^&^ The Y strain of *Trypanosoma cruzi* is known to be partially sensitive to benznidazole given by oral route for 20 days at 100 mg/kg (~50% efficacy) at classical protocol in mice [33].

**Table 4:** Pharmacokinetic parameters of oral and subcutaneous (implant) administration of benznidazole during treatment with the same total dose (100 mg/animal) in healthy and in *T. cruzi*- infected mice.

Parameter	Unit	Treatments and Formulations			
		Oral Not Infected	Oral Infected	Subcutaneous Implant Not Infected	Subcutaneous Implant Infected
*C*_max_	µg/mL	1.5718	1.0444	14.6115	30.7782
*C_last obs_*	µg/mL	0.6886	1.0374	0.6712	0.4558
*C_last pred_*	µg/mL	0.7481	nd	0.6169	0.3134
*T*_max_	h	360	696	24	24
*AUC*_0-t_	µg/mL.h	1185.022	755.789	2471.565	3737.566
MRT_0-t_	h	616.99	531.76	349.74	220.69
Cl/F	L/h	0.0484	nd	0.0339	0.0255
*k_e_*	1/h	0.000783	nd	0.001405	0.002492

For oral treatment, each mouse received oral gavage with a 2.5 mg dose/day for 40 days (25 ± 1.5 g bodyweight mice). The implants were inserted in SC tissue at day 0. nd: not possible to calculate with the mathematical model. n = 3 for each group. Cl/F: the apparent clearance. Non-compartmental analysis was the best fit for plasma concentration–time profiles after extravascular input. AUC*_0-t_* was estimated using a trapezoidal linear up/Log down method using PK solver 2.0 add-in software [38].

## Data Availability

The data presented in this study are available on request from the corresponding author.

## Supplementary Materials

The following supporting information can be downloaded at: https://www.mdpi.com/article/10.3390/pharmaceutics15041126/s1, Figure S1. X-ray diffraction patterns of benznidazole. Figure S2. X-ray diffraction patterns of benznidazole refined by the Rietveld method (black), using the crystallographic data described by Soares-Sobrinho et al. (2008) [18] (orange). Figure S3. X-ray diffraction patterns of PCL implant (without drug).
