# Supplementary material for: Poly-ε-Caprolactone Implants for Benznidazole Prolonged Release: An Alternative to Chagas Disease Oral Treatment
Source: Pharmaceutics. 2023 Apr 2;15(4):1126. doi: 10.3390/pharmaceutics15041126 (PMC10147077; doi:10.3390/pharmaceutics15041126)
Supplement: Supplementary file 1 [file pharmaceutics-15-01126-s001.zip › pharmaceutics-2143097-supplementary.pdf]

Supplementary Information

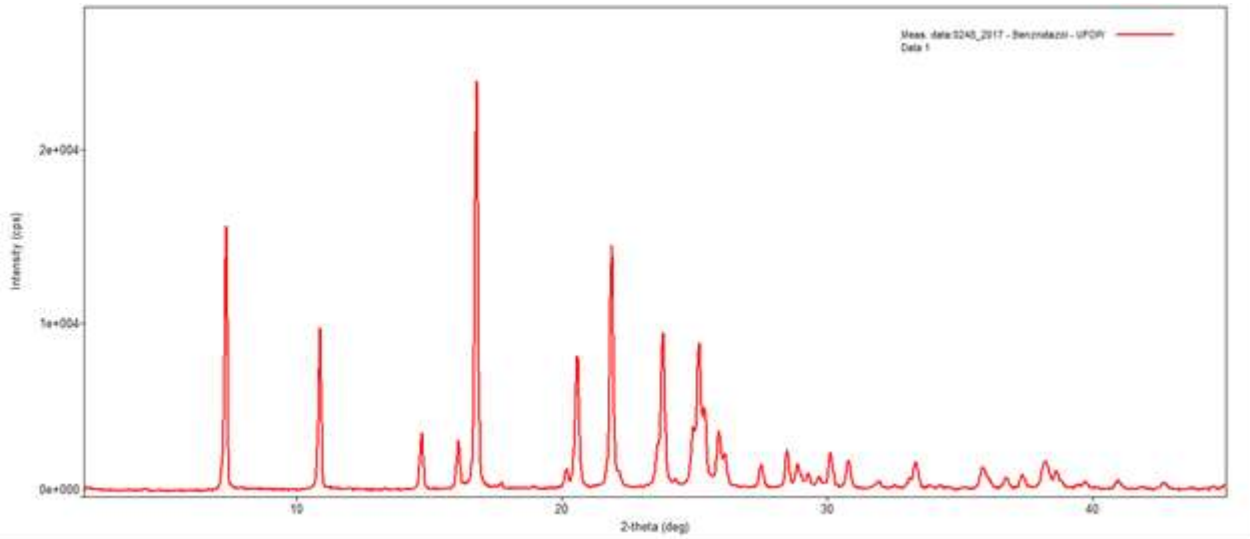

Figure I: X-ray diffraction patterns of benznidazole.

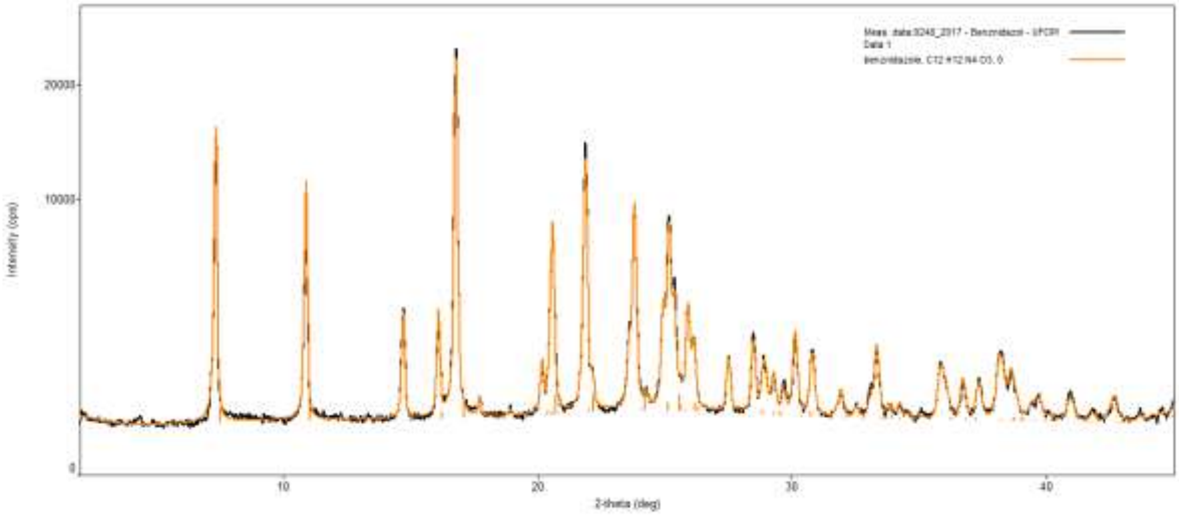

Figure II: X-ray diffraction patterns of benznidazole refined by the *Rietveld* method (black), using the crystallographic data described by Soares-Sobrinho et al. (2008) [18] (orange).

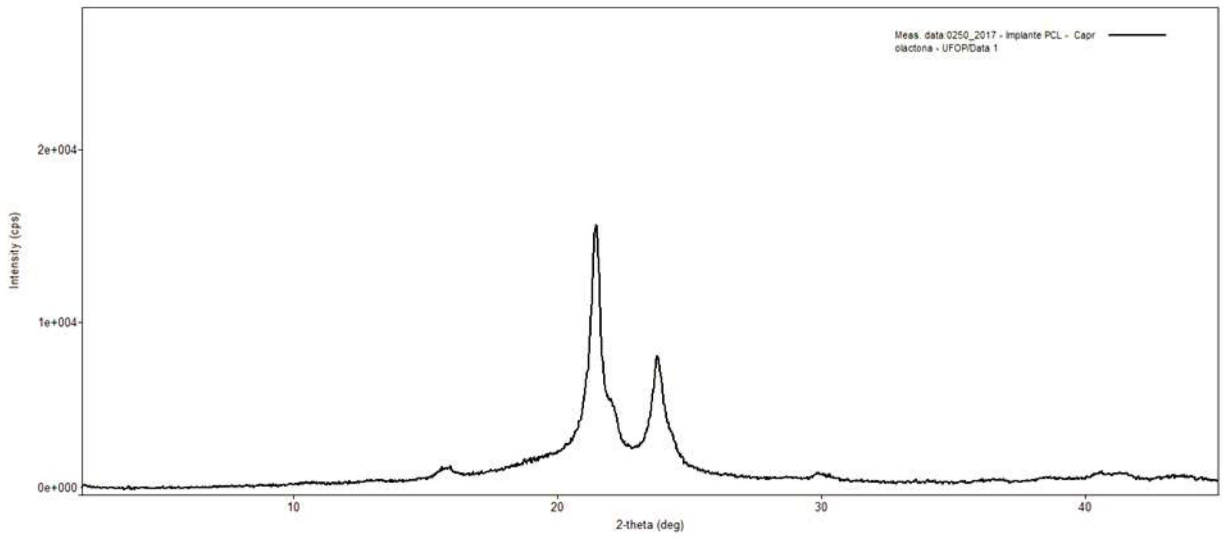

Figure III: X-ray diffraction patterns of PCL implant (without drug)
